# Supplementary material for: Determinants of early initiation of first antenatal care visit in Ethiopia based on the 2019 Ethiopia mini-demographic and health survey: A multilevel analysis
Source: PLoS One. 2023 Mar 6;18(3):e0281038. doi: 10.1371/journal.pone.0281038 (PMC9987803; doi:10.1371/journal.pone.0281038)
Supplement: Attachment — Submitted filename: Comments and Suggestions_ANC_Plos One.docx [file pone.0281038.s001.docx]

**Comments**

**General view:**

I would say the authors have submitted an important piece of work in its field of relevance. However, I have few suggestions for the authors to improve this manuscript.

**Title**

The topic is unattractive. I suggest modification to make the title catchier, attractive and simple.

The title has some repetitions. It should be only multilevel not multilevel mixed effect

The authors said “2019 Ethiopia mini demographic health survey” which is not the right name of survey

**Suggested Title “Determinants of early initiation of first antenatal care visit in Ethiopia based on the 2019 Ethiopia mini-demographic and health survey: A multilevel Analysis”**

**Abstract**

**Method:** The authors should be more specific and "in order" in describing the methods. Determinants, associated factors, risk factors, please be specific throughout the document. which one do you think is appropriate considering your study design?

“A multilevel mixed logistic regression model” needs to rewritten in a correct way

**Introduction**

There are a lot of editorial errors in this section needs to be rewritten.

The author stated that *“However, those previous studies in Ethiopia were conducted in specific areas with small sample sizes, mainly facility-based and were not nationally representative.”* Is this really the case? There are many previous studies conducted with sample sizes and nationally **representative (Based on EDHS 2016 may be one example)**. Hence, I suggest the authors to elaborate more as to why this study is important and what will be the novel contribution of this study.

The gap to this study should be clear. Thus, authors need to convince readers that this is additional information.

Determinant or factors associated please be consistent with this terminology throughout your document. Is it possible to use both terminologies interchangeably?

**Methods**

The author stated “2019 intermediate Ethiopian mini demography and health survey” is this the correct name of the survey? Please be consistent throughout the document

The way you define your source and study population were seeming incorrect. Please re define your source and study population.

The author stated *“in the bivariable two-level binary logistic regression were candidates for multivariable multilevel logistic regression analysis.”* Two-level or multi-level you wrote as the two were different and needs to be re-written

Sample size determination was not clear. On what criteria does the final sample size was considered.

**Results**

Add horizontal line in table, because of your data type it is difficult to see which number corresponding to which variable currently

There are a lot of editorial errors in your result. For instance, (the age range of 20-29 (55.2%) years old., 2,691 (91.7) were married, etc) please work on that

**Discussion**

The authors should discuss something on the existing policy or programme that target ANC services, and how these findings can be useful for improving the existing programme.

The authors should explore more on the relationship between variable. The authors should also show the implications of the results in more informative and practical way.

The authors should also follow guideline of the journal for in text and list of references

The manuscript has several grammatical and typological flaws that hamper its readability.

Align with this journal manuscript submission guideline

I reserve any further comments until the authors have substantially revised or justified their comments.
