# Supplementary material for: Determinants of early initiation of first antenatal care visit in Ethiopia based on the 2019 Ethiopia mini-demographic and health survey: A multilevel analysis
Source: PLoS One. 2023 Mar 6;18(3):e0281038. doi: 10.1371/journal.pone.0281038 (PMC9987803; doi:10.1371/journal.pone.0281038)
Supplement: Attachment — Submitted filename: Response to reviewers.docx [file pone.0281038.s003.docx]

**Author response to reviewers**

**Dear Editor and Reviewers,**

Thank you very much for your email dated 27^th^ December 2022 incorporating the insight of the editor and reviewer’s comments. We, the authors, would like to express our gratitude to you for the insightful and constructive review that has led to the great improvement of our paper entitled **“Determinants of early initiation of first antenatal care visit in Ethiopia based on 2019 the Ethiopia mini-demographic and health survey: A multilevel Analysis”.** We have carefully reviewed the comments given by the reviewer and revised the manuscript accordingly. Our responses are given in a point-by-point manner for the reviewers' comments using the Author’s response to the reviewer form. If you have any concerns to be addressed, we are happy to consider them.

Best regards!

**Version 1:** PONE-D-22-13822R1

Date: 12/29/2022

**Reviewer #1 comments and an author response**

**Comments**

**Reviewers' comment #1:** There are a lot of editorial errors in this section that needs to be rewritten. For instance, “As far as the researcher search of pieces of literature concerned, the is no research assessing the determinants of early initiation…” I suggest the authors to elaborate more as to why this study is important and what will be the novel contribution of this study.

**Author’s Response:** Thank you very much dear reviewer for this valuable comment. We, the authors, accept the comment and the correction has been made accordingly (*See pages 6-7, lines 121 – 138*). The importance and novel contribution of this study has been justified as “However, most studies in Ethiopia were conducted in specific areas with small sample sizes, mainly facility-based, and were not nationally representative. Moreover, the previous studies mainly emphasized on individual-level determinants with little attention given to community-level determinants. However, this could underscore the importance of considering contextual determinants when designing appropriate antenatal care service strategies. Thus, for bridging all those gaps, this study used a recent national-level data (2019 Ethiopia Mini Demographic and Health Survey data) to determine the current magnitude and individual and community-level determinants of early initiation of the first antenatal care visit among reproductive-age women. Therefore, the findings of this study will be invaluable to identify the determinants that impede the early initiation of first ANC visits as a result respective programs are made more appropriately towards the identified determinants and will also have paramount importance to show country-level figures, and screen out modified and persistent determinants, which in turn maximize the timely initiation of the first ANC visits, and achievement of the SDG #3 of eliminating maternal and neonatal mortality by 2030.”
